# Comparative Analysis of Osteoarthritis Therapeutics: A Justification for Harnessing Retrospective Strategies via an Inverted Pyramid Model Approach

**DOI:** 10.3390/biomedicines12112469

**Published:** 2024-10-28

**Authors:** Quinn T. Ehlen, Jacob Jahn, Ryan C. Rizk, Thomas M. Best

**Affiliations:** 1 University of Miami Miller School of Medicine, Miami, FL 33136, USA; rcr109@miami.edu (R.C.R.); txb440@miami.edu (T.M.B.); 2 Department of Orthopedics, University of Miami, Miami, FL 33124, USA; 3 UHealth Sports Medicine Institute, University of Miami, Miami, FL 33124, USA

**Keywords:** osteoarthritis, drug development, OA, OA treatment, KOA

## Abstract

In this review, we seek to explore two distinct approaches to the clinical management of OA: a prospective approach, addressing primarily one’s genetic predisposition to OA and generating early intervention options, and the retrospective approach, aimed at halting or reversing OA progression post-symptom onset. The clinical management of OA remains challenging, largely due to the limited availability of preventative treatments and failure of existing therapies to modify or reverse the underlying pathophysiology. The prospective approach involves the identification of genetic markers associated with OA and utilizes in vitro and in vivo models to characterize the underlying disease mechanism. Further, this approach focuses on identifying genetic predispositions and unique molecular subtypes of OA to develop individualized treatment plans based on patient genotypes. While the current literature investigating this strategy has been notable, this approach faces substantial challenges, such as extensive time burdens and utilization of extensive genetic testing that may not be economically feasible. Additionally, there is questionable justification for such extensive investigations, given OA’s relatively low mortality rates and burden when contrasted with diseases like specific forms of cancer, which rely heavily on the prospective approach. Alternatively, the retrospective approach primarily focuses on intervention following symptom onset and aims to utilize novel therapeutics to slow or reverse the inflammatory cascade typically seen in disease progression. These treatments, like Hippo pathway inhibitors, have shown initial promise in halting OA progression and alleviating OA symptomology by modulating cellular processes to preserve articular cartilage. In comparison to the prospective approach, the retrospective strategy is likely more cost-effective, more widely applicable, and does not necessitate thorough and invasive genetic screening. However, this approach must still be weighed against the typical natural history of disease progression, which frequently results in total knee arthroplasty and unacceptable outcomes for 15–20% of patients. From a comparative analysis of these two approaches, this review argues that the retrospective strategy, with ideally lower time and economic burden and greater accessibility, offers a more reasonable and effective solution in the context of OA management. Using a similar approach to other management of chronic diseases, we suggest an “Inverted Pyramid” model algorithm, a structured research and development regimen that prioritizes generating widely effective therapies first, with subsequent refinement of treatments based on the development of patient resistance to these therapies. We argue that this strategy may reduce the need for total knee arthroplasty while improving patient outcomes and accessibility.

## 1. Introduction

Osteoarthritis (OA) is widely recognized as the most common form of arthritis, impacting over 500 million patients globally and affecting over 45% of individuals 65 or older in the United States alone [1,2,3]. The pathophysiology of OA, resembling that of other diseases of inflammatory origin, is largely due to the degeneration of joint cartilage and surrounding bone, which stimulates an inflammatory cascade, ultimately causing patient symptoms [4,5]. However, OA is not limited to cartilage degradation alone; rather, it is a whole-joint disease that involves multiple joint tissues, including the infrapatellar fat pad, synovial membrane, subchondral bone, and periarticular structures. Degradation of these neighboring and surrounding structures leads to an array of symptomology, ranging from mild, ambient pain, stiffness, or swelling to more severe impairment of overall quality of life, such as ambulatory or activity hindrance and debilitating loss of one’s range of motion [6,7]. Globally, the impact of OA is substantial, not only due to its high disease prevalence but also due to its significant impairment of patient quality of life and contribution to functional disability, particularly among older adults [8,9]. These factors cause a significant economic impact, resulting in an estimated annual loss of nearly USD 45 billion in the US primarily from medical treatment and reduced workplace productivity [10]. Additionally, due to the sedentary lifestyle often imparted upon patients with OA, there are strong correlations between the onset of OA symptoms and the development of concurrent conditions that may lead to more severe consequences and negative health outcomes [11].

Despite its prevalence and widely characterized negative impact on overall patient health [8], current OA management centers primarily on symptom management rather than reversing or modifying the underlying molecular signatures of the disease [12]. In knee OA (KOA), treatment regimens include the use of NSAIDs, corticosteroids, hyaluronic acid (HA) injections, and orthobiologics such as PRP and stem cell therapies, yet frequently culminate in total knee arthroplasty (TKA) should symptoms persist or increase in severity [13,14,15,16]. While there are various reports on the efficacy of the current treatments available for KOA [13,14,17,18], alternative approaches are necessary in order to improve upon the current treatment regimens, as each option possesses notable risks and limitations. Due to the existing shortcomings in OA therapy and management, we propose a novel approach based on an “Inverted Pyramid” model, which focuses on the development of broad, effective therapies targeting the underlying mechanisms of OA, followed by refinement of these treatments for more specific subgroups as needed.

In this review, we categorize current clinical approaches to OA management as either prospective or retrospective. We define a prospective approach to KOA management as strategies that focus on preclinical intervention and, ideally, prevention of the disease by targeting specific genetic and molecular markers, while our retrospective approach refers to therapies that aim to halt or reverse disease progression after symptom onset, independent of underlying genetic subtypes. We will explore these various approaches in detail by evaluating their respective benefits, limitations, and potential roles in the future of KOA management.

## 2. Challenges in OA Management

Prior to a discussion on the current approaches to OA management, it is important to review the challenges associated with the clinical picture of KOA pathophysiology and current treatment strategies. At its core, there is a central issue in modern KOA management, as preventative strategies largely revolve around lifestyle modifications, including maintenance of a healthy weight, engaging in moderate-intensity exercise, and avoiding traumatic joint injuries [19]. While crucial, these interventions are oftentimes insufficient to prevent the onset of KOA, as lifestyle modifications are often not sufficiently met [20], and little can be done to mitigate one’s trauma risk [21]. Additionally, lifestyle modifications focusing on decreasing the effects of joint loading are becoming increasingly quieted, as more recent research points to an increased role of different genetic markers in predisposing an individual to OA onset, largely independent from contributory mechanical factors [22,23,24].

In today’s treatment landscape, pharmacological prevention of disease focused on the underlying etiology of KOA remains an unmet need in clinical management. While there have been robust efforts to develop drugs that could prevent or delay the onset of KOA, such as therapeutics targeting inflammatory pathways or cartilage metabolism [25,26], these have not yet reached a point by which they are effective and cost-effective to justify their usage in a clinical setting. Understandably, OA pathophysiology is exceedingly complex due to an interaction of mechanical, genetic, biochemical, and environmental factors, which each possess their own specific challenges [27,28]. Specifically, mechanical stress and abnormal joint loading, such as those induced by obesity or joint instability, result in disrupted cartilage homeostasis and lead to upregulated catabolic activity in chondrocytes as well as increased breakdown of proteoglycans and collagen [28]. This is further exacerbated by proinflammatory cytokines, which accelerate extracellular matrix breakdown and further deteriorate joint integrity. Therefore, research and therapeutic development in OA is forced to focus on targeting a single aspect of disease pathogenesis to create effective treatments for preventing OA onset.

In addition to the limited capacity for therapies to prevent KOA onset, there is also inconsistency in outcomes for treatments currently utilized to treat symptomatic KOA. For example, while HA injections are hypothesized to improve joint lubrication and reduce friction, their effectiveness in mitigating disease progression is minimal and their overall effectiveness in OA symptom management is often debated [15].

Opposed to the therapies aimed at relieving general symptoms are Disease-Modifying Osteoarthritis Drugs (DMOADs), which, as their name implies, seek to alter the underlying molecular pathways contributing to OA pathogenesis. While these pathways have become an increasingly significant focus of research [25], progress has remained elusive in their translation to clinical phenotypes and more personalized treatment algorithms.

In addition to the complex, multifactorial nature of OA, which makes identification of a targetable element for DMOADs challenging, there are additional roadblocks in the drug development process. KOA, while inflicting a vast burden on patients in terms of prevalence and incidence of lifestyle-altering symptoms [29], carries an exceedingly low mortality rate and overall risk profile [30]. As opposed to therapies aimed at treating higher mortality diseases, such as various types of cancer, DMOADs designed for the treatment of OA must be effective at providing symptom relief while independent of genetic profiling of patients. This is due to the fact that, at present, the cost and economic feasibility of genetic sequencing to identify molecular subtypes may outweigh the benefit received from said drugs. Current genetic sequencing protocols used for the identification of rare diseases may range from USD 5000 to 20,000 [31,32], and there is no established precedent for this approach in OA. Furthermore, TKA, an effective retrospective treatment option, already exists and is used widely in clinical practice. TKA is both widely effective and relatively cost-effective, especially when compared to the cost of genetic profiling [33]. However, there remains debate over the effectiveness of TKA versus nonsurgical management of KOA [34]. This could be due to insurance providers often requiring patients to undergo nonoperative treatments for a certain period before covering the cost of TKA. For example, the Centers for Medicare & Medicaid Services require a history of nonoperative management of at least 3 months as a criterion for prior authorization of TKA coverage [35]. Therefore, any new OA therapy must not only match or exceed the efficacy and cost of TKA but also must provide additional benefits, such as avoiding the well-documented risks associated with surgery [36].

Therefore, DMOADs and other therapies focused on OA management are silently called upon to not only be effective at alleviating OA symptoms and reversing OA pathophysiology but also must do so without the use of widespread genetic profiling to allow for a lower cost than TKA. Despite the challenge of developing a widely effective therapy without the use of genetic identification techniques, we will explore the progress that has been made to date. However, a debate is necessary to compare whether a retrospective or prospective approach is most suitable to meet the demand for an alternative to the current regimen for OA treatment.

## 3. Prospective Approach to OA

Herein, we define the prospective approach to OA treatment as the utilization of molecular sequencing techniques to recognize and intervene in patients demonstrated to be at higher risk of developing OA. This strategy relies heavily on targeting the underlying molecular and genetic contributors to OA, with the goal of preventing or significantly delaying disease onset. Therefore, identifying genetic mutations within OA pathogenesis that may predict OA susceptibility is vital to this approach. For instance, recent studies have found that mutations in *GDF5* alter its role in cartilage development [37,38]. In addition to *GDF5*, studies have also identified mutations in the gene *MCF2L*, which is involved in nerve growth, as additional contributors to OA susceptibility [39,40]. Further, molecular markers such as *GLIS3,* a transcriptional regulator involved in cartilage homeostasis, and *DOT1L*, a histone methyltransferase involved in the regulation of bone formation, have also been recognized as contributing factors to increasing OA susceptibility [41,42]. Research identifying targetable elements in OA pathophysiology suggests that inhibiting harmful genetic variants could potentially slow disease progression. We will now discuss these, amongst other potential prospective therapeutic approaches that have shown promise to prevent OA onset and progression.

### 3.1. Identification of Genetic and Molecular Markers

Due to significant advances in genetic and molecular sequencing techniques, numerous genetic markers and molecular subtypes have been identified as contributors to OA pathogenesis. Among the most widely characterized are genetic mutations in the *GDF5* gene [43]. In research conducted by Zhang and colleagues, aberrations in *GDF5* were identified, particularly in the regulatory regions of the gene, which have been associated with an increased risk of KOA, as well as OA in other locations [37]. From the findings in Zhang’s investigation, *GDF5* has emerged at the forefront of studies targeting the underlying physiology of OA. Further, since its identification, it has served as a model for the recognition of other genetic mutations and spurred increased investment into characterizing targets that can modulate their expression.

*MCF2L* is also recognized as a significant genetic marker involved in OA pathogenesis [23], which encodes a guanine nucleotide exchange factor that is involved in physiologic nerve growth and repair [40]. Day-Williams et al. investigated the influence of *MCF2L* and, like *GDF5*, polymorphisms in *MCF2L* were linked to OA susceptibility, particularly in the knee [40]. More specifically, the role of *MCF2L* in nerve growth suggests that it may contribute to the pain and sensory changes observed in OA, making it a potential target for therapies aimed at modulating nerve-related symptoms.

In addition to the aforementioned investigations characterizing individual genes, larger-scale genome-wide association studies (GWAS) are underway, such as one conducted by the arcOGEN Consortium. Importantly, these wide-scale studies have identified numerous other loci associated with risk for OA, such as the *SMAD3* gene, which encodes a protein within the TGF-β signaling cascade [44,45,46]. TGF-β is an integral inflammatory molecule that is closely involved in the maintenance of cartilage homeostasis; therefore, dysregulation of this pathway has been demonstrated to be correlated to both the initiation and progression of inflammatory diseases, including OA [47].

Recent studies have also indicated *GLIS3* as a key transcriptional factor for chondrocyte differentiation and matrix production [41]. Casalone et al. demonstrated that the variant rs10116772 in the intron of *GLIS3* was significantly associated with OA, particularly in individuals who underwent total joint replacement due to severe OA [41]. While its exact mechanisms in OA are still unclear, the literature suggests that the role of this gene in chondrocyte differentiation could influence cartilage health and overall OA progression.

In addition to the aforementioned genetic markers, disruptor of telomeric silencing 1-like (*DOT1L*) is an epigenetic regulator that encodes a histone H3 lysine 79 methyltransferase which has been shown to play a role in endochondral bone formation [48]. The SNP rs12982744 within *DOT1L* has been associated with a reduction in minimum joint space width (minJSW), a key indicator of hip OA, suggesting that genetic variation in *DOT1L* affects cartilage and bone structure [42]. In large-scale studies, particularly in male subjects, rs12982744 reached genome-wide significance by demonstrating a 17% increased association with hip OA [42]. The study found that *DOT1L* men had a larger minJSW and a stronger association with hip OA, while the prevalence of OA increased in women post-menopause, suggesting that sex hormones may play a role in its progression.

### 3.2. Characterization of Molecular Subtypes

OA is increasingly recognized as a heterogeneous disease with different molecular subtypes that may require distinct therapeutic approaches [49]. For example, patients with a largely inflammatory component to their OA have elevated levels of proinflammatory cytokines, including IL-1β and TNF-α, in their synovial fluid [50]. These cytokines can initiate and amplify inflammatory cascades within the joint leading to synovitis, cartilage breakdown, and eventual joint destruction. Through this process, NF-κB is activated and upregulates the expression of matrix metalloproteinases (MMPs) and aggrecanases, enzymes responsible for the degradation of cartilage extracellular matrix components like collagen and aggrecan [51]. Therefore, patients with this subtype may benefit from therapies that specifically target expression of these cytokines or their upstream signaling cascades [52]. Conversely, OA in patients with metabolic syndrome involves different molecular mechanisms from those seen in inflammatory OA. Its pathogenesis has been implicated to stem from insulin resistance and lipid metabolism dysregulation [53,54]. Accordingly, identifying their underlying mechanisms via molecular profiling could drive the development of personalized therapies that target the specific causes of OA in these particular patient populations.

Epigenetic modifications, such as DNA methylation and histone modification, have also been shown to play a significant role in the pathogenesis of OA [55]. Such modifications influence gene expression by altering the accessibility of transcriptional machinery to specific regions of the genome without changing the DNA sequence itself. For instance, research conducted by Jin et al. demonstrated that altered methylation patterns in genes involved in cartilage development and degradation have been observed in OA patients [56]. Specifically, hypermethylation of anabolic genes like *SOX9*, a transcription factor important for chondrogenesis, can downregulate cartilage repair processes, while hypomethylation of catabolic genes such as *MMP13* can lead to excessive cartilage breakdown [57]. These epigenetic changes can influence gene expression without altering the underlying DNA sequence, contributing to the complexity of OA and offering another layer of potential therapeutic targets [58]. Numerous other studies and research groups are currently investigating genetic and epigenetic factors and their association with OA, as seen in Table 1.

### 3.3. Prospective Approach Challenges

Despite the progress made to date in understanding genetic contributors and molecular underpinnings of OA, it is integral to translate these findings into clinical practice. However, this remains a significant challenge and an active topic of debate. One major hurdle is the aforementioned complexity of OA pathogenesis, which makes it difficult to develop a widely applicable, “one-size-fits-all” therapy. Additionally, variability in genetic expression between individuals with OA means that a therapy targeting a specific genetic pathway involved in its progression may only be effective in an isolated subset of patients. An example of this is seen in breast cancer treatment, where therapies targeting the HER2 protein are only effective in patients whose tumors overexpress the HER2 gene [59]. Therefore, while the identification of genetic markers provides a foundation for developing patient-specific targeted therapies, the clinical implementation of these strategies will require careful consideration of both the patient’s overall molecular and clinical profile *prior to symptom onset*. Seeing as the vast majority of patients with OA do not likely present for medical treatment until symptoms such as pain and stiffness are present, identification of molecular subtypes prior to symptom onset presents a practical and economic challenge. Furthermore, the cost of such testing may limit its accessibility, particularly in healthcare systems with limited resources. As a result, while the prospective approach offers promising avenues for preventing or mitigating OA, its application in routine clinical practice may be restricted to specific high-risk populations or settings with the necessary resources and genome sequencing infrastructure.

**Table 1:** Current research on genetic markers associated with increased OA risk along with the associated single-nucleotide polymorphism variants.

Gene	Association with OA	GWAS SNP	Author, Year	Study Type	Study Focus	Ref.
GDF5	Promotes differentiation and proliferation of mesenchymal stem cells into chondrocytes.	rs143383	Zhang et al., 2015	Meta-analysis	Association between GDF5 polymorphisms and increased risk of OA in multiple joints.	[37]
			Takahata et al., 2022	Review	Regulatory mechanisms of GDF5 expression in cartilage	[38]
			Sun et al., 2021	Review	Growth differentiation factor 5 as a therapeutic candidate for OA.	[43]
MCF2L	Involved in nerve growth and regeneration in joint tissues.	rs11842874	Day-Williams et al., 2011	GWAS	MCF2L polymorphism association with knee OA susceptibility.	[40]
			Shepherd et al., 2015	Expression Study	Expression analysis of MCF2L genetic susceptibility locus’s role in OA.	[39]
SMAD3	Regulates cartilage development and repair through TGF-beta signaling.	rs1290137, rs12901071	Zhang et al., 2018	GWAS	Association between SMAD3 polymorphisms and knee OA with TGF-β signaling pathways.	[44]
			Yang et al., 2018	Polymorphism study	SMAD3 gene polymorphisms’ role in cartilage homeostasis and OA progression.	[45]
GLIS3	Promotes chondrocyte differentiation and matrix production.	rs1011677, rs10974438	Lv et al., 2021	Molecular classification study	GLIS3 as a transcriptional regulator in cartilage homeostasis.	[49]
DOT1L	Methylates histone H3K79 to regulate genes important for cartilage formation and maintenance.	rs12982744	Chapman et al., 2012	GWAS	Association between DOT1L polymorphisms and reduced joint space width.	[23]
			Lv et al., 2021	GWAS and functional study	DOT1L as an important regulator of bone and cartilage.	[49]
TGFB1	Regulates articular chondrocyte hypertrophy and matrix degradation by promoting expression of Mmp13 and Adamts5.	rs75621460	Rice et al., 2021	GWAS	Functional impact of the SNP rs75621460 near TGFB1 on gene expression and DNA methylation in cartilage and synovium.	[60]
MGP	Inhibits cartilage calcification.	rs4764133	den Hollander et al., 2017	GWAS and functional study	Genetic variants associated with hand OA.	[61]
PLEC	Encodes a cytoskeletal protein that maintains tissue integrity.	rs11780978	Rice et al., 2019	GWAS	Methylation quantitative trait loci associated with OA risk alleles.	[62]
			Sorial et al., 2020	Functional genomics study	Impact of PLEC knockdown on cellular pathways related to OA.	[63]
RUNX2	Important for osteoblastic differentiation and skeletogenesis.	rs10948172	Rice et al., 2018	Association studies	SNP rs10948172 affects DNA methylation and gene expression at RUNX2 and SUPT3H.	[64]
ALDH1A2	Encodes an enzyme important role for retinoic acid synthesis.	rs12915901	Shepherd et al., 2018	GWAS	Decreased expression of ALDH1A2 affects chondrogenic markers which increases hand OA risk.	[65]
RWDD2B	Involved in a multi-tissue methylation-expression quantitative trait locus associated with the SNP rs6516886.	rs6516886	Parker et al., 2020	Functional genetic and epigenetic analysis	rs6516886 increases methylation at CpG site cg20220242 and reduces RWDD2B expression.	[66]
COLGALT2	Important for collagen fibril stability.	rs11583641	Kehayova et al., 2023	Functional genomics study	SNP rs11583641 decreases COLGALT2 expression in cartilage.	[67]

## 4. Retrospective Approach to OA

Pivoting to an alternative strategy, we define the retrospective approach as one that focuses on treating OA after the onset of symptoms, with a similar goal to halt or reverse disease progression. This approach may be especially applicable to patients who are not candidates for TKA, such as those with comorbidities or advanced age, and for those who are experiencing significant symptoms such as severe joint pain that limits daily activity, leading to weight gain, etc. Unlike prospective strategies that require genetic or molecular profiling, retrospective approaches target the broad mechanisms of OA that are common across most patients. Accordingly, this approach may be a more practical and cost-effective option for disease management on a large scale.

### 4.1. Current Retrospective Approaches

In current clinical practice, HA and steroid injections are widely used retrospective treatments that aim to reduce pain and inflammation in OA patients. These therapies are based on the understanding that inflammation plays a central role in OA progression and, by controlling its expression, symptoms can be alleviated [68,69]. While HA is not typically used as a first-line therapy for OA, it is a safe and effective treatment with minimal side effects, such as a few days of pain and swelling [70]. Moreover, it has demonstrated significant short-term efficacy (under six months) in relieving knee OA pain [71]. Intra-articular CS injections have also demonstrated efficacy in improving function and reducing pain in the early phase post-administration; however, there remains debate regarding their long-term safety. Concerns have been raised by McAlindon et al. and other studies, suggesting that IA CS injections may potentially cause cartilage harm [14,72]. In regards to the efficacy of both HA and CS injections, Rodriguez-Merchan et al. concluded that, while CS provided the greatest pain relief in the first four weeks after injection, HA produced better results at 5 and 13 weeks, with effects lasting up to 26 weeks [73]. However, the limitations of these treatments are well documented (Table 2) [13,14,17,18]. Accordingly, there is an unmet need for the development of more effective retrospective treatments that can modify the underlying disease rather than current strategies that emphasize symptom management.

When discussing retrospective therapies, it is paramount to mention the most established approach for end-stage knee OA, joint arthroplasty. As a retrospective strategy, TKA is highly effective in the majority of patients after severe progression of symptom onset and has been demonstrated to provide significant pain relief and restoration of joint function [74]. It is currently considered the gold standard for patients with severe symptoms and loss of quality of life, particularly in cases where conservative therapies have failed. In a prospective study conducted by Chaudhary et al., the functional and clinical outcomes of TKA in 40 patients with osteoarthritis were evaluated using the Knee Society Score (KSS) and postoperative mechanical axis alignment [75]. The study demonstrated a significant improvement in KSS, with 57.4% of patients achieving excellent scores and the average KSS increasing from 177 to 225 points post-surgery. The findings confirmed TKA led to favorable long-term outcomes in treating end-stage OA. However, despite its effectiveness and the majority of patients reporting high satisfaction, in a retrospective cohort [76] where 67.5% were very satisfied, 15% to 25% of patients remained dissatisfied with the outcomes of primary TKA [77]. Overall, while TKA is not perfect and has its limitations, the success of TKA in improving the quality of life for OA patients has set a high benchmark for emerging therapies. To be considered viable alternatives, new treatments must not only match the efficacy of arthroplasty but also offer additional benefits, such as reducing the risks and recovery time associated with surgery.

Recently, the Hippo pathway has emerged as a potential target for retrospective OA treatment [78,79]. In the recent literature, the Hippo pathway has been characterized to be involved in regulating cell growth and apoptotic events; therefore, its dysregulation has been implicated in various inflammatory diseases, including cancer and OA [80,81]. Investigations characterizing the effects of Hippo pathway inhibition in inflammatory diseases have been promising [82,83], suggesting investigation in patients with KOA.

Hippo pathway inhibitors, which target and inhibit the activity of key proteins, including YAP/TAZ, reduce inflammation, preserve cartilage, and promote tissue repair in in vitro investigations [78]. For example, in models of post-traumatic OA (PTOA), Hippo inhibitors have been shown to inhibit chondrocyte apoptosis and significantly decrease cartilage degradation [79]. Furthermore, Gong et al. investigated YAP inhibition in a mouse model and found that YAP siRNA significantly reduced proinflammatory responses and catabolic activity while promoting anabolism [84]. Although this study and others exploring Hippo pathway inhibitors were conducted in mouse models, the results show promising potential for future clinical applications in OA treatment. This is especially relevant since Hippo pathway inhibitors target inflammatory proteins present in all individuals, rather than specific mutations; Hippo pathway inhibitors are an attractive option for retrospective treatment. Additionally, targeting a fundamental pathway involved in OA pathogenesis demonstrates that these inhibitors could potentially benefit a wide range of patients, regardless of their specific genetic background or stage of disease. However, while this is a promising retrospective treatment for OA, explicit safety evaluations are sparse and have yet to be answered [85].

**Table 2:** Current research on retrospective treatment approaches for osteoarthritis, mechanisms of action, advantages, and shortcomings of these approaches.

Treatment Approach	Mechanism of Action	Advantages	Shortcomings	Ref
TKA	Knee joint replaced with artificial implants.	Long-term cost-effectiveness, pain relief, joint function, success rate, long-terms results.	Approximately 20% patient dissatisfaction post-TKA due to unmet expectations, recovery time, and surgical complications.	[16,18,36,74,76,77,86,87]
Intra-articular Hyaluronic Acid (HA) Injections	Binds chondrocyte and synoviocyte receptors stimulating production of endogenous HA.	Improves joint lubrication, provides improved mobility/pain relief, minimally invasive.	Temporary effects, does not reverse cartilage damage.	[15,17,68,88,89,90,91,92]
Corticosteroid (CS) Injections	Blocks production of proinflammatory cytokines and suppresses cartilage degrading enzymes.	Provides quick and effective short-term pain relief and improvement in joint function.	Temporary effects, repeated injections may diminish in effectiveness over time.	[14,69,89,91]
Platelet-Rich Plasma Injections	Releases growth factors such as PDGF and TGF-β [69,93].	Delivers concentrated growth factors and potentially improves pain/function.	Variable effectiveness across patients and often requires multiple treatments.	[69,91,93,94]
Stem Cell Injections	MSCs which can develop into cartilage-producing cells and reduce inflammation.	Inflammation reduction, cartilage regeneration, and joint function improvement.	Limited clinical data on its ability to regenerate cartilage or stop disease progression.	[95,96,97]
Non-steroidal anti-inflammatory drugs (NSAIDs)	Inhibits enzymes responsible for inflammation and pain in the joints.	Reduces inflammation, lowers the production of pro-inflammatory prostaglandins and cytokines.	Gastric ulcers and bleeding, increased cardiovascular risks, particularly with long-term use.	[13,98,99,100,101,102]
Hippo Pathway Inhibitors	Promotes the activity of YAP/TAZ which helps to prevent chondrocyte apoptosis.	Potential to reduce inflammation and regenerate cartilage through promotion of chondrocyte survival.	Long-term efficacy/safety unknown, high cost, and limited availability.	[78,79,80,81,82]
RANKL Inhibitor (Denosumab)	Inhibits RANKL	Slows down erosive joint progression and promotes bone remodeling.	Improvements in pain and function often take up to 96 weeks, potential bone health complications after long-term use or treatment cessation.	[103]

### 4.2. Retrospective Approach Benefits

Importantly, one of the key advantages of the retrospective approach is its ability to provide broadly applicable treatments that do not require extensive genetic or molecular testing. Accordingly, this strategy should lead to a more practical and accessible option for the majority of OA patients who may not have access to advanced diagnostic tools.

The cost-effectiveness of retrospective treatments represents another potentially significant advantage. By focusing on common mechanisms of disease, such as inflammation and cartilage degradation, these treatments can be widely implemented at a lower cost than personalized therapies. This is particularly important in managing a chronic and widespread condition like OA, where the economic burden on healthcare systems is substantial and rising. Additionally, this alleviates the cost-imposed burden that OA therapeutics face, as retrospective approaches may be able to compete, in terms of both efficacy and affordability, with TKA without the risks associated with surgical intervention.

## 5. A Proposed Model for Translation to OA Management

As mentioned previously, OA treatment is currently constrained by two key principles. First, the existence of a highly effective retrospective treatment, TKA, sets a standard for any new therapy. TKA is not only widely effective but also relatively cost-effective compared to other interventions discussed in this review [104]. Ideally, for any new treatment to be viable, it must not only rival the success of TKA but also provide unique benefits, particularly for those patients with surgical contraindications.

Secondly, despite the high prevalence, morbidity, and societal burden of OA, the disease is associated with a relatively low risk of mortality. This low mortality rate makes it difficult to justify the extensive use of genetic sequencing or molecular characterization to identify specific OA subtypes for targeted treatment. Unlike high-mortality conditions such as cancer, where genetic profiling is essential for treatment planning, the cost and practicality of such an approach in OA are questionable.

### Inverted Pyramid Model

Based on the concepts discussed above, we propose the use of a model for OA therapy we term the “Inverted Pyramid” model. This approach is somewhat analogous to the evolution of cancer treatment regimens, where broadly effective therapies are often developed first, followed by more targeted treatments for patients who do not respond to initial interventions. For instance, this model has been successfully applied in the treatment of chronic lymphocytic leukemia (CLL), which is a reasonable comparison for OA within the realm of cancer, as it highly impacts quality of life with relatively low mortality risk [105].

**Figure 1:**
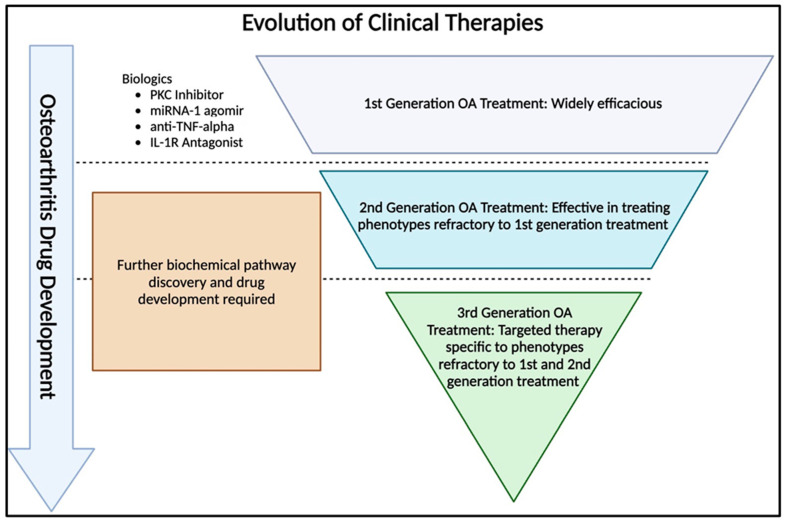
Proposed inverted pyramid model approach for OA therapy development.

To expand on this idea, we will briefly discuss the development of CLL therapeutics as a paradigm to harness in OA therapeutic development [106]. In CLL, the initial development of first-generation covalent BTK inhibitors provided a broadly effective treatment for a large portion of patients, ranging from 50 to 85% [106,107]. However, some patients either became or were inherently refractory to this treatment, leading to the development of noncovalent BTK inhibitors [108,109], which addressed a smaller subset of refractory patients. As research continued, even more targeted treatments, such as BTK degraders [110], were developed to treat those who were refractory to both first- and second-generation treatments. The proposed stepwise refinement of therapy illustrates the value of starting with broadly applicable treatments before focusing in on specific molecular underpinnings, as illustrated in Figure 1.

It is the opinion of the authors that OA treatment could benefit from a similar approach, focusing first on developing broadly effective retrospective therapies that target common mechanisms of disease, such as inflammation and cartilage degradation, after symptom onset. These treatments can then be further refined based on patient subtypes, specifically those who did not respond well to initial therapies. Rather than characterizing the molecular endotype of every OA patient, we suggest focusing on those that either do not respond to treatment or may present with worsened symptomology. This will help identify genetic markers that indicate a higher risk of poor prognosis. We also propose that, while widely applicable, this intervention will be best suited for patients who present with OA early in disease progression and minimal radiographic changes (Kellgren-Lawrence grades 1–2). To simplify, this approach is analogous to the ease of searching for a red marble in a jar of blue marbles rather than searching for the same red marble in a much larger pool of blue marbles. It ensures that the majority of patients receive effective treatment while also allowing for the gradual refinement of therapies as more is uncovered about the pathogenesis of OA.

## 6. Retrospective vs. Prospective Approach

Building on the discussions of both prospective and retrospective strategies raised within this review, it becomes evident that each approach offers unique advantages while facing distinct challenges. While prospective treatments focus on early intervention and the prevention of OA onset through targeted genetic and molecular approaches, retrospective therapies aim to intervene after symptom onset, ideally offering the potential to halt or even reverse disease progression. It is essential to consider how both of these approaches align with current clinical needs and their potential to improve patient outcomes in a landscape where TKA remains the benchmark for end-stage OA treatment.

### 6.1. Effectiveness in Patient Outcomes

Retrospective treatments, such as Hippo pathway inhibitors, present a promising opportunity based on preclinical studies to enhance patient outcomes by targeting one of several potential mechanisms driving OA progression. Unlike many current therapies that primarily manage symptoms, these treatments offer the dual advantage of symptom relief and potentially altering the disease’s trajectory, representing a meaningful advancement in OA therapy. While prospective approaches aim to prevent the onset of OA or slow its progression before clinical symptoms manifest, their efficacy is often hindered by the complexities of early diagnosis and the necessity for highly targeted interventions. In contrast, retrospective approaches are likely to be more broadly applicable and can be immediately deployed to address the needs of a wider patient population, making them a more practical solution in many clinical settings.

### 6.2. Cost and Feasibility

From an economic aspect, the retrospective approach stands out for its cost-effectiveness, as it does not demand the extensive and expensive infrastructure required for genetic testing and the implementation of personalized medicine. These features should make the retrospective approach a more viable option for large-scale adoption, particularly in resource-limited environments [104,111]. Although prospective strategies contribute valuable insights into underlying disease mechanisms, they come with significant costs, primarily due to the need for genetic screening and the development of individualized treatment regimens, which, at present, may not be supported by insurance providers. Therefore, these financial and logistical burdens may limit their use, especially for a condition like OA, which has a lower mortality risk compared to life-threatening diseases such as cancer, where genetic sequencing is more common.

### 6.3. Proposed Treatment Strategy

As outlined in our discussion of the “Inverted Pyramid” model, we advocate for a treatment strategy in OA that prioritizes the development of broad, widely applicable retrospective therapies capable of effectively treating the majority of patients. These foundational therapies could form the cornerstone of OA management, much like the first-line treatments used in other chronic diseases such as hypertension and diabetes [112]. However, it is important to recognize that, while we propose an increased focus on retrospective therapies, achieving a baseline efficacy level that justifies their use over TKA will require substantial effort. Additionally, in order to identify targetable elements within OA pathogenesis, it is apparent that prospective-based research interventions may be necessary to identify common elements in OA pathogenesis amongst a wide base of the patient population, regardless of molecular endotype. Further, these therapies must demonstrate comparable or superior effectiveness to TKA, particularly for patients who may not be suitable candidates for surgery.

Once a broadly effective treatment is in place, subsequent research should focus on identifying and addressing the mechanisms of resistance in specific patient subgroups. This phased approach enables the gradual refinement of treatment options, ensuring that even patients who do not initially respond to therapy receive effective care. This strategy mirrors what was previously described as an “Inverted Pyramid” model used in CLL research, where broadly effective therapies are initially discovered and developed, followed by more refined and targeted treatments for patients who do not respond to initial interventions.

## Abbreviations

DMOAD	Disease-Modifying Osteoarthritis Drug
DOT1L	Disruptor of Telomeric Silencing 1-Lik
GLIS3	GLIS Family Zinc Finger 3
GWAS	Genome-Wide Association Studies
HA	Hyaluronic Acid
Hippo	Hippo Signaling Pathway
IL-1β	Interleukin-1 Beta
KOA	Knee Osteoarthritis
MMP	Matrix Metalloproteinase
minJSW	minimum joint space width
NSAIDs	Non-Steroidal Anti-Inflammatory Drugs
OA	Osteoarthritis
PRP	Platelet-Rich Plasma
RANKL	Receptor Activator of Nuclear Factor Kappa-B Ligand
RNAi	RNA Interference
SNP	Single-Nucleotide Polymorphism
TGF-β	Transforming Growth Factor Beta
TKA	Total Knee Arthroplasty
TNF-α	Tumor Necrosis Factor Alpha
Wnt	Wingless-related Integration Site (Pathway)

## References

[1] Yang G., Wang J., Liu Y., Lu H., He L., Ma C., Zhao Z. (2023). Burden of knee osteoarthritis in 204 countries and territories, 1990–2019: Results from the Global Burden of Disease Study 2019. Arthritis Care Res..

[2] Nelson A.E., Hu D., Arbeeva L., Alvarez C., Cleveland R.J., Schwartz T.A., Murphy L.B., Helmick C.G., Callahan L.F., Renner J.B. (2021). The Prevalence of Knee Symptoms, Radiographic, and Symptomatic Osteoarthritis at Four Time Points: The Johnston County Osteoarthritis Project, 1999–2018. ACR Open Rheumatol..

[3] Wen C., Xiao G. (2022). Advances in osteoarthritis research in 2021 and beyond. J. Orthop. Transl..

[4] Chow Y.Y., Chin K.-Y. (2020). The role of inflammation in the pathogenesis of osteoarthritis. Mediat. Inflamm..

[5] Molnar V., Matišić V., Kodvanj I., Bjelica R., Jeleč Ž., Hudetz D., Rod E., Čukelj F., Vrdoljak T., Vidović D. (2021). Cytokines and chemokines involved in osteoarthritis pathogenesis. Int. J. Mol. Sci..

[6] Yan H., Guo J., Zhou W., Dong C., Liu J. (2022). Health-related quality of life in osteoarthritis patients: A systematic review and meta-analysis. Psychol. Health Med..

[7] Davis A., King L., Stanaitis I., Hawker G. (2022). Fundamentals of osteoarthritis: Outcome evaluation with patient-reported measures and functional tests. Osteoarthr. Cartil..

[8] Steinmetz J.D., Culbreth G.T., Haile L.M., Rafferty Q., Lo J., Fukutaki K.G., Cruz J.A., Smith A.E., Vollset S.E., Brooks P.M. (2023). Global, regional, and national burden of osteoarthritis, 1990–2020 and projections to 2050: A systematic analysis for the Global Burden of Disease Study 2021. Lancet Rheumatol..

[9] Scheuing W.J., Reginato A.M., Deeb M., Acer Kasman S. (2023). The burden of osteoarthritis: Is it a rising problem?. Best. Pract. Res. Clin. Rheumatol..

[10] Zhao X., Shah D., Gandhi K., Wei W., Dwibedi N., Webster L., Sambamoorthi U. (2019). Clinical, humanistic, and economic burden of osteoarthritis among noninstitutionalized adults in the United States. Osteoarthr. Cartil..

[11] Lee J., Chang R.W., Ehrlich-Jones L., Kwoh C.K., Nevitt M., Semanik P.A., Sharma L., Sohn M.W., Song J., Dunlop D.D. (2015). Sedentary behavior and physical function: Objective evidence from the Osteoarthritis Initiative. Arthritis Care Res..

[12] Oo W.M., Yu S.P.-C., Daniel M.S., Hunter D.J. (2018). Disease-modifying drugs in osteoarthritis: Current understanding and future therapeutics. Expert. Opin. Emerg. Drugs.

[13] Da Costa B.R., Pereira T.V., Saadat P., Rudnicki M., Iskander S.M., Bodmer N.S., Bobos P., Gao L., Kiyomoto H.D., Montezuma T. (2021). Effectiveness and safety of non-steroidal anti-inflammatory drugs and opioid treatment for knee and hip osteoarthritis: Network meta-analysis. bmj.

[14] Najm A., Alunno A., Gwinnutt J.M., Weill C., Berenbaum F. (2021). Efficacy of intra-articular corticosteroid injections in knee osteoarthritis: A systematic review and meta-analysis of randomized controlled trials. Jt. Bone Spine.

[15] Colen S., Van Den Bekerom M.P., Mulier M., Haverkamp D. (2012). Hyaluronic acid in the treatment of knee osteoarthritis: A systematic review and meta-analysis with emphasis on the efficacy of different products. BioDrugs.

[16] Katz J.N. (2006). Total joint replacement in osteoarthritis. Best. Pract. Res. Clin. Rheumatol..

[17] Bannuru R.R., Vaysbrot E.E., Sullivan M.C., McAlindon T.E. (2014). Relative efficacy of hyaluronic acid in comparison with NSAIDs for knee osteoarthritis: A systematic review and meta-analysis. Semin. Arthritis Rheum..

[18] Kane R.L., Saleh K.J., Wilt T.J., Bershadsky B. (2005). The functional outcomes of total knee arthroplasty. J. Bone Jt. Surg..

[19] O’Reilly S., Doherty M. (2001). Lifestyle changes in the management of osteoarthritis. Best. Pract. Res. Clin. Rheumatol..

[20] Spitaels D., Vankrunkelsven P., Desfosses J., Luyten F., Verschueren S., Van Assche D., Aertgeerts B., Hermens R. (2017). Barriers for guideline adherence in knee osteoarthritis care: A qualitative study from the patients’ perspective. J. Eval. Clin. Pract..

[21] Whittaker J., Runhaar J., Bierma-Zeinstra S., Roos E. (2021). A lifespan approach to osteoarthritis prevention. Osteoarthr. Cartil..

[22] Aubourg G., Rice S., Bruce-Wootton P., Loughlin J. (2022). Genetics of osteoarthritis. Osteoarthr. Cartil..

[23] Chapman K., Valdes A.M. (2012). Genetic factors in OA pathogenesis. Bone.

[24] Ratneswaran A., Kapoor M. (2021). Osteoarthritis year in review: Genetics, genomics, epigenetics. Osteoarthr. Cartil..

[25] Cho Y., Jeong S., Kim H., Kang D., Lee J., Kang S.-B., Kim J.-H. (2021). Disease-modifying therapeutic strategies in osteoarthritis: Current status and future directions. Exp. Mol. Med..

[26] Jiang P., Hu K., Jin L., Luo Z. (2024). A brief review of current treatment options for osteoarthritis including disease-modifying osteoarthritis drugs (DMOADs) and novel therapeutics. Ann. Med. Surg..

[27] He Y., Li Z., Alexander P.G., Ocasio-Nieves B.D., Yocum L., Lin H., Tuan R.S. (2020). Pathogenesis of osteoarthritis: Risk factors, regulatory pathways in chondrocytes, and experimental models. Biology.

[28] Guilak F. (2011). Biomechanical factors in osteoarthritis. Best. Pract. Res. Clin. Rheumatol..

[29] Leifer V.P., Katz J.N., Losina E. (2022). The burden of OA-health services and economics. Osteoarthr. Cartil..

[30] Veronese N., Cereda E., Maggi S., Luchini C., Solmi M., Smith T., Denkinger M., Hurley M., Thompson T., Manzato E. (2016). Osteoarthritis and mortality: A prospective cohort study and systematic review with meta-analysis. Semin. Arthritis Rheum..

[31] Incerti D., Xu X.-M., Chou J.W., Gonzaludo N., Belmont J.W., Schroeder B.E. (2022). Cost-effectiveness of genome sequencing for diagnosing patients with undiagnosed rare genetic diseases. Genet. Med..

[32] Schwarze K., Buchanan J., Fermont J.M., Dreau H., Tilley M.W., Taylor J.M., Antoniou P., Knight S.J., Camps C., Pentony M.M. (2020). The complete costs of genome sequencing: A microcosting study in cancer and rare diseases from a single center in the United Kingdom. Genet. Med..

[33] Losina E., Walensky R.P., Kessler C.L., Emrani P.S., Reichmann W.M., Wright E.A., Holt H.L., Solomon D.H., Yelin E., Paltiel A.D. (2009). Cost-effectiveness of total knee arthroplasty in the United States: Patient risk and hospital volume. Arch. Intern. Med..

[34] Pacheco-Brousseau L., Abdelrazeq S.Y., Kelly S.E., Pardo J.P., Dervin G., Nahar N., Stacey D., Wells G.A. (2023). Total and partial knee arthroplasty versus non-surgical interventions of the knee for moderate to severe osteoarthritis. Cochrane Database Syst. Rev..

[35] Nin D.Z., Chen Y.W., Talmo C.T., Hollenbeck B.L., Mattingly D.A., Niu R., Chang D.C., Smith E.L. (2022). Costs of Nonoperative Procedures for Knee Osteoarthritis in the Year Prior to Primary Total Knee Arthroplasty. J. Bone Jt. Surg. Am..

[36] Belmont Jr P.J., Goodman G.P., Waterman B.R., Bader J.O., Schoenfeld A.J. (2014). Thirty-day postoperative complications and mortality following total knee arthroplasty: Incidence and risk factors among a national sample of 15,321 patients. J. Bone Jt. Surg..

[37] Zhang R., Yao J., Xu P., Ji B., Luck J.V., Chin B., Lu S., Kelsoe J.R., Ma J. (2015). A comprehensive meta-analysis of association between genetic variants of GDF5 and osteoarthritis of the knee, hip and hand. Inflamm. Res..

[38] Takahata Y., Hagino H., Kimura A., Urushizaki M., Yamamoto S., Wakamori K., Murakami T., Hata K., Nishimura R. (2022). Regulatory mechanisms of Prg4 and Gdf5 expression in articular cartilage and functions in osteoarthritis. Int. J. Mol. Sci..

[39] Shepherd C., Skelton A.J., Rushton M.D., Reynard L.N., Loughlin J. (2015). Expression analysis of the osteoarthritis genetic susceptibility locus mapping to an intron of the MCF2L gene and marked by the polymorphism rs11842874. BMC Med. Genet..

[40] Day-Williams A.G., Southam L., Panoutsopoulou K., Rayner N.W., Esko T., Estrada K., Helgadottir H.T., Hofman A., Ingvarsson T., Jonsson H. (2011). A variant in MCF2L is associated with osteoarthritis. Am. J. Hum. Genet..

[41] Casalone E., Tachmazidou I., Zengini E., Hatzikotoulas K., Hackinger S., Suveges D., Steinberg J., Rayner N.W., Wilkinson J.M., Panoutsopoulou K. (2018). A novel variant in GLIS3 is associated with osteoarthritis. Ann. Rheum. Dis..

[42] Evangelou E., Valdes A.M., Castano-Betancourt M.C., Doherty M., Doherty S., Esko T., Ingvarsson T., Ioannidis J.P., Kloppenburg M., Metspalu A. (2013). The DOT1L rs12982744 polymorphism is associated with osteoarthritis of the hip with genome-wide statistical significance in males. Ann. Rheum. Dis..

[43] Sun K., Guo J., Yao X., Guo Z., Guo F. (2021). Growth differentiation factor 5 in cartilage and osteoarthritis: A possible therapeutic candidate. Cell Prolif..

[44] Zhang L., Zhang L., Zhang H., Wang W., Zhao Y. (2018). Association between SMAD3 gene rs12901499 polymorphism and knee osteoarthritis in a Chinese population. J. Clin. Lab. Anal..

[45] Yang H.-Y., Hu W.-H., Jiang T., Zhao H. (2018). SMAD3 gene rs12901499 polymorphism increased the risk of osteoarthritis. Biosci. Rep..

[46] (2012). Identification of new susceptibility loci for osteoarthritis (arcOGEN): A genome-wide association study. Lancet.

[47] Duan M., Wang Q., Liu Y., Xie J. (2021). The role of TGF-β2 in cartilage development and diseases. Bone Jt. Res..

[48] Castaño Betancourt M.C., Cailotto F., Kerkhof H.J., Cornelis F.M., Doherty S.A., Hart D.J., Hofman A., Luyten F.P., Maciewicz R.A., Mangino M. (2012). Genome-wide association and functional studies identify the DOT1L gene to be involved in cartilage thickness and hip osteoarthritis. Proc. Natl. Acad. Sci. USA.

[49] Lv Z., Yang Y.X., Li J., Fei Y., Guo H., Sun Z., Lu J., Xu X., Jiang Q., Ikegawa S. (2021). Molecular Classification of Knee Osteoarthritis. Front. Cell Dev. Biol..

[50] Wang T., He C. (2018). Pro-inflammatory cytokines: The link between obesity and osteoarthritis. Cytokine Growth Factor Rev..

[51] Mukherjee A., Das B. (2024). The role of inflammatory mediators and matrix metalloproteinases (MMPs) in the progression of osteoarthritis. Biomater. Biosyst..

[52] Liu S., Deng Z., Chen K., Jian S., Zhou F., Yang Y., Fu Z., Xie H., Xiong J., Zhu W. (2022). Cartilage tissue engineering: From proinflammatory and anti-inflammatory cytokines to osteoarthritis treatments. Mol. Med. Rep..

[53] Rojas-Rodríguez J., Escobar-Linares L.E., Garcia-Carrasco M., Escárcega R.O., Fuentes-Alexandro S., Zamora-Ustaran A. (2007). The relationship between the metabolic syndrome and energy-utilization deficit in the pathogenesis of obesity-induced osteoarthritis. Med. Hypotheses.

[54] Zhuo Q., Yang W., Chen J., Wang Y. (2012). Metabolic syndrome meets osteoarthritis. Nat. Rev. Rheumatol..

[55] Miranda-Duarte A. (2018). DNA methylation in osteoarthritis: Current status and therapeutic implications. Open Rheumatol. J..

[56] Jin L., Ma J., Chen Z., Wang F., Li Z., Shang Z., Dong J. (2023). Osteoarthritis related epigenetic variations in miRNA expression and DNA methylation. BMC Med. Genom..

[57] Neefjes M., van Caam A.P.M., van der Kraan P.M. (2020). Transcription Factors in Cartilage Homeostasis and Osteoarthritis. Biology.

[58] Grandi F.C., Bhutani N. (2020). Epigenetic therapies for osteoarthritis. Trends Pharmacol. Sci..

[59] Slamon D.J., Leyland-Jones B., Shak S., Fuchs H., Paton V., Bajamonde A., Fleming T., Eiermann W., Wolter J., Pegram M. (2001). Use of chemotherapy plus a monoclonal antibody against HER2 for metastatic breast cancer that overexpresses HER2. N. Engl. J. Med..

[60] Rice S.J., Roberts J.B., Tselepi M., Brumwell A., Falk J., Steven C., Loughlin J. (2021). Genetic and Epigenetic Fine-Tuning of TGFB1 Expression Within the Human Osteoarthritic Joint. Arthritis Rheumatol..

[61] den Hollander W., Boer C.G., Hart D.J., Yau M.S., Ramos Y.F.M., Metrustry S., Broer L., Deelen J., Cupples L.A., Rivadeneira F. (2017). Genome-wide association and functional studies identify a role for matrix Gla protein in osteoarthritis of the hand. Ann. Rheum. Dis..

[62] Rice S.J., Tselepi M., Sorial A.K., Aubourg G., Shepherd C., Almarza D., Skelton A.J., Pangou I., Deehan D., Reynard L.N. (2019). Prioritization of PLEC and GRINA as Osteoarthritis Risk Genes Through the Identification and Characterization of Novel Methylation Quantitative Trait Loci. Arthritis Rheumatol..

[63] Sorial A.K., Hofer I.M.J., Tselepi M., Cheung K., Parker E., Deehan D.J., Rice S.J., Loughlin J. (2020). Multi-tissue epigenetic analysis of the osteoarthritis susceptibility locus mapping to the plectin gene PLEC. Osteoarthr. Cartil..

[64] Rice S.J., Aubourg G., Sorial A.K., Almarza D., Tselepi M., Deehan D.J., Reynard L.N., Loughlin J. (2018). Identification of a novel, methylation-dependent, RUNX2 regulatory region associated with osteoarthritis risk. Hum. Mol. Genet..

[65] Shepherd C., Zhu D., Skelton A.J., Combe J., Threadgold H., Zhu L., Vincent T.L., Stuart P., Reynard L.N., Loughlin J. (2018). Functional Characterization of the Osteoarthritis Genetic Risk Residing at ALDH1A2 Identifies rs12915901 as a Key Target Variant. Arthritis Rheumatol..

[66] Parker E., Hofer I.M.J., Rice S.J., Earl L., Anjum S.A., Deehan D.J., Loughlin J. (2021). Multi-Tissue Epigenetic and Gene Expression Analysis Combined With Epigenome Modulation Identifies RWDD2B as a Target of Osteoarthritis Susceptibility. Arthritis Rheumatol..

[67] Kehayova Y.S., Wilkinson J.M., Rice S.J., Loughlin J. (2023). Osteoarthritis genetic risk acting on the galactosyltransferase gene COLGALT2 has opposing functional effects in articulating joint tissues. Arthritis Res. Ther..

[68] Bowman S., Awad M.E., Hamrick M.W., Hunter M., Fulzele S. (2018). Recent advances in hyaluronic acid based therapy for osteoarthritis. Clin. Transl. Med..

[69] Irshad S., Waleed U., Zafar M.H., Ramzan M.T., Tariq M.A., Hassan M., Sohaib M.A., Liaquat S., Mehmood S., Ali R.S. (2024). The Efficacy of Intra-articular Platelet-Rich Plasma Injection Versus Corticosteroid Injection in the Treatment of Knee Osteoarthritis: A Prospective Comparative Analysis. Cureus.

[70] Chavda S., Rabbani S.A., Wadhwa T. (2022). Role and Effectiveness of Intra-articular Injection of Hyaluronic Acid in the Treatment of Knee Osteoarthritis: A Systematic Review. Cureus.

[71] Altman R., Hackel J., Niazi F., Shaw P., Nicholls M. (2018). Efficacy and safety of repeated courses of hyaluronic acid injections for knee osteoarthritis: A systematic review. Semin. Arthritis Rheum..

[72] McAlindon T.E., LaValley M.P., Harvey W.F., Price L.L., Driban J.B., Zhang M., Ward R.J. (2017). Effect of Intra-articular Triamcinolone vs Saline on Knee Cartilage Volume and Pain in Patients With Knee Osteoarthritis: A Randomized Clinical Trial. Jama.

[73] Rodriguez-Merchan E.C. (2013). Intra-articular Injections of Hyaluronic Acid and Other Drugs in the Knee Joint. HSS J..

[74] Goh G.S., Liow M.H.L., Tay Y.W.A., Chen J.Y., Xu S., Pang H.-N., Tay D.K.-J., Chia S.-L., Lo N.-N., Yeo S.-J. (2020). The long-term impact of preoperative psychological distress on functional outcomes, quality of life, and patient satisfaction after total knee arthroplasty: A ten-year follow-up study. Bone Jt. J..

[75] Chaudhary C., Kothari U., Shah S., Pancholi D. (2024). Functional and Clinical Outcomes of Total Knee Arthroplasty: A Prospective Study. Cureus.

[76] Walker L.C., Clement N.D., Bardgett M., Weir D., Holland J., Gerrand C., Deehan D.J. (2018). The WOMAC score can be reliably used to classify patient satisfaction after total knee arthroplasty. Knee Surg. Sports Traumatol. Arthrosc..

[77] Rodriguez-Merchan E.C. (2021). Patient Satisfaction Following Primary Total Knee Arthroplasty: Contributing Factors. Arch. Bone Jt. Surg..

[78] Sun K., Guo J., Guo Z., Hou L., Liu H., Hou Y., He J., Guo F., Ye Y. (2023). The roles of the Hippo-YAP signalling pathway in cartilage and osteoarthritis. Ageing Res. Rev..

[79] Cai X., Warburton C., Perez O.F., Wang Y., Ho L., Finelli C., Ehlen Q.T., Wu C., Rodriguez C.D., Kaplan L. (2024). Hippo-PKCζ-NFκB signaling axis: A druggable modulator of chondrocyte responses to mechanical stress. Iscience.

[80] Pan D. (2022). The unfolding of the Hippo signaling pathway. Dev. Biol..

[81] Fu M., Hu Y., Lan T., Guan K.-L., Luo T., Luo M. (2022). The Hippo signalling pathway and its implications in human health and diseases. Signal Transduct. Target. Ther..

[82] Dey A., Varelas X., Guan K.-L. (2020). Targeting the Hippo pathway in cancer, fibrosis, wound healing and regenerative medicine. Nat. Rev. Drug Discov..

[83] Yeung Y.T., Aziz F., Guerrero-Castilla A., Arguelles S. (2018). Signaling pathways in inflammation and anti-inflammatory therapies. Curr. Pharm. Des..

[84] Gong Y., Li S.J., Liu R., Zhan J.F., Tan C., Fang Y.F., Chen Y., Yu B. (2019). Inhibition of YAP with siRNA prevents cartilage degradation and ameliorates osteoarthritis development. J. Mol. Med..

[85] Kakiuchi-Kiyota S., Schutten M.M., Zhong Y., Crawford J.J., Dey A. (2019). Safety Considerations in the Development of Hippo Pathway Inhibitors in Cancers. Front. Cell Dev. Biol..

[86] Steinhaus M.E., Christ A.B., Cross M.B. (2017). Total Knee Arthroplasty for Knee Osteoarthritis: Support for a Foregone Conclusion?. HSS J..

[87] Chang J., Fu M., Cao P., Ding C., Wang D. (2022). Patient-Reported Quality of Life Before and After Total Knee Arthroplasty: A Multicenter Observational Study. Patient Prefer. Adherence.

[88] Goh S.L., Chong M.W., Ling J., Jaafar Z., Lim Z.L., Yau M.Y., Ong T., Richards J. (2024). Semi-invasive therapies for pain in knee osteoarthritis: A systematic review and network meta-analysis. Pain Pract..

[89] Golovachev N., Ghayyad K., Sarli N., Meade J., Hirsch D., Kachooei A.R. (2024). Nonoperative Management of Trapeziometacarpal Joint Arthritis: A Systematic Review of the Clinical Trials. Cureus.

[90] Park J.G., Sim J., Han S.B. (2024). Association between intra-articular hyaluronic acid injections in delaying total knee arthroplasty and safety evaluation in primary knee osteoarthritis: Analysis based on Health Insurance Review and Assessment Service (HIRA) claim database in Republic of Korea. BMC Musculoskelet. Disord..

[91] Bensa A., Sangiorgio A., Boffa A., Salerno M., Moraca G., Filardo G. (2024). Corticosteroid injections for knee osteoarthritis offer clinical benefits similar to hyaluronic acid and lower than platelet-rich plasma: A systematic review and meta-analysis. EFORT Open Rev..

[92] Moreland L.W. (2003). Intra-articular hyaluronan (hyaluronic acid) and hylans for the treatment of osteoarthritis: Mechanisms of action. Arthritis Res. Ther..

[93] Khuba S., Khetan D., Kumar S., Garg K.K., Gautam S., Mishra P. (2023). Role of platelet rich plasma in management of early knee osteoarthritis pain: A retrospective observational study. Interv. Pain Med..

[94] Chen P., Huang L., Ma Y., Zhang D., Zhang X., Zhou J., Ruan A., Wang Q. (2019). Intra-articular platelet-rich plasma injection for knee osteoarthritis: A summary of meta-analyses. J. Orthop. Surg. Res..

[95] Shi X., Chen H., Yang H., Xue S., Li Y., Fang X., Ding C., Zhu Z. (2024). Aptamer-Modified Tetrahedral Framework Nucleic Acid Synergized with TGF-β3 to Promote Cartilage Protection in Osteoarthritis by Enhancing Chondrogenic Differentiation of MSCs. ACS Appl. Mater. Interfaces.

[96] Wu J., Wu J., Xiang W., Gong Y., Feng D., Fang S., Wu Y., Liu Z., Li Y., Chen R. (2024). Engineering exosomes derived from TNF-α preconditioned IPFP-MSCs enhance both yield and therapeutic efficacy for osteoarthritis. J. Nanobiotechnol..

[97] Giorgino R., Alessandri Bonetti M., Migliorini F., Nannini A., Vaienti L., Peretti G.M., Mangiavini L. (2024). Management of hip osteoarthritis: Harnessing the potential of mesenchymal stem cells—A systematic review. Eur. J. Orthop. Surg. Traumatol..

[98] Miyake Y., Mitani S., Namba Y., Umehara N., Kawamoto T., Furuichi S. (2024). Efficacy of S-Flurbiprofen Plaster for Analgesia Following Total Hip Arthroplasty. Cureus.

[99] Kraev K., Uchikov P., Hristov B., Kraeva M., Basheva-Kraeva Y., Doykov M., Popova-Belova S., Geneva-Popova M. (2024). Exploring the impact of curcumin on osteoarthritis symptomatology: Correlations and insights from a Bulgarian cohort. Folia Med..

[100] Neogi T., Dell’Isola A., Englund M., Turkiewicz A. (2024). Frequent use of prescription NSAIDs among people with knee or hip osteoarthritis despite contraindications to or precautions with NSAIDs. Osteoarthr. Cartil..

[101] Cioroianu G.O., Florescu A., Simionescu C.E., Sas T.N., Tarniţă D.N., Rogoveanu O.C. (2024). The therapeutic benefits of NSAIDs and physical therapy in knee osteoarthritis. Rom. J. Morphol. Embryol..

[102] Ospina J., Carmona J.U., López C. (2024). Short-Term Effects of Two COX-2 Selective Non-Steroidal Anti-Inflammatory Drugs on the Release of Growth Factors and Cytokines from Canine Platelet-Rich Gel Supernatants. Gels.

[103] Wittoek R., Verbruggen G., Vanhaverbeke T., Colman R., Elewaut D. (2024). RANKL blockade for erosive hand osteoarthritis: A randomized placebo-controlled phase 2a trial. Nat. Med..

[104] Kamaraj A., To K., Seah K.M., Khan W.S. (2020). Modelling the cost-effectiveness of total knee arthroplasty: A systematic review. J. Orthop..

[105] Ammann E.M., Shanafelt T.D., Wright K.B., McDowell B.D., Link B.K., Chrischilles E.A. (2018). Updating survival estimates in patients with chronic lymphocytic leukemia or small lymphocytic lymphoma (CLL/SLL) based on treatment-free interval length. Leuk. Lymphoma.

[106] Chirino A., Montoya S., Safronenka A., Taylor J. (2023). Resisting the Resistance: Navigating BTK Mutations in Chronic Lymphocytic Leukemia (CLL). Genes.

[107] Tam C.S., Brown J.R., Kahl B.S., Ghia P., Giannopoulos K., Jurczak W., Šimkovič M., Shadman M., Österborg A., Laurenti L. (2022). Zanubrutinib versus bendamustine and rituximab in untreated chronic lymphocytic leukaemia and small lymphocytic lymphoma (SEQUOIA): A randomised, controlled, phase 3 trial. Lancet Oncol..

[108] Montoya S., Thompson M.C. (2023). Non-Covalent Bruton’s Tyrosine Kinase Inhibitors in the Treatment of Chronic Lymphocytic Leukemia. Cancers.

[109] Sharma S., Galanina N., Guo A., Lee J., Kadri S., Van Slambrouck C., Long B., Wang W., Ming M., Furtado L.V. (2016). Identification of a structurally novel BTK mutation that drives ibrutinib resistance in CLL. Oncotarget.

[110] Montoya S., Bourcier J., Noviski M., Lu H., Thompson M.C., Chirino A., Jahn J., Sondhi A.K., Gajewski S., Tan Y.S. (2024). Kinase-impaired BTK mutations are susceptible to clinical-stage BTK and IKZF1/3 degrader NX-2127. Science.

[111] Christensen K.D., Dukhovny D., Siebert U., Green R.C. (2015). Assessing the Costs and Cost-Effectiveness of Genomic Sequencing. J. Pers. Med..

[112] Flood D., Edwards E.W., Giovannini D., Ridley E., Rosende A., Herman W.H., Jaffe M.G., DiPette D.J. (2023). Integrating hypertension and diabetes management in primary health care settings: HEARTS as a tool. Rev. Panam. Salud Pública.

